# Osteoblast responses to implant materials and the role of vitamin D in dental implant osseointegration: a narrative review

**DOI:** 10.1186/s40729-026-00723-0

**Published:** 2026-10-22

**Authors:** Ioanna Arvanitaki, Bilal Al-Nawas, Desiree Gül, Aya Khamis

**Affiliations:** 1 Department of Oral and Maxillofacial Surgery, Plastic Surgery, University Medical Centre of the Johannes Gutenberg-University, Mainz, Germany; 2 Department of Otorhinolaryngology Head and Neck Surgery, Molecular and Cellular Oncology, University Medical Centre, 55131 Mainz, Germany; 3 Oral Pathology Department, Faculty of Dentistry, Alexandria University, Alexandria, Egypt

**Keywords:** Dental implants, Osseointegration, Osteoblasts, Implant materials, Titanium, Zirconia, Titanium-zirconium alloy, Vitamin D, Bone healing, Translational medicine

## Abstract

**Purpose:**

This narrative review aims to summarise current evidence on osteoblast responses to commonly used dental implant materials and to evaluate the potential role of vitamin D in modulating biological processes involved in dental implant osseointegration.

**Methods:**

A narrative literature review was performed using PubMed/MEDLINE, Google Scholar, and reference lists of relevant articles and reviews. The literature was last updated in September 2026. The review focused on implant materials and surface characteristics, osteoblast adhesion, proliferation, differentiation, mineralised matrix formation, vitamin D biology, and clinical evidence related to vitamin D and dental implant osseointegration.

**Results:**

Current evidence indicates that implant surface characteristics, including roughness, topography, surface chemistry, hydrophilicity, and coatings, influence osteoblast behaviour and the biological response at the bone-implant interface. Titanium remains the most established implant material, while zirconia and titanium-zirconium alloys have shown favourable biological and clinical performance in selected indications. Vitamin D is known to be biologically relevant for bone metabolism and osteoblast activity, and experimental studies suggest that it may support osteogenic differentiation and mineralised matrix formation. However, current human clinical evidence regarding vitamin D and implant outcomes remains limited and heterogeneous.

**Conclusions:**

Osseointegration is influenced by a complex interaction between implant material characteristics, surface properties, osteoblast responses, systemic vitamin D status, and host-related biological factors. Although vitamin D is known to be biologically relevant and experimental findings are promising, there is currently insufficient evidence to show that vitamin D has variable effects on osseointegration around titanium, zirconia, or titanium-zirconium implants. Further in vitro, in vivo, and clinical studies are needed using clearly defined implant materials, surface characteristics, vitamin D status or supplementation protocols, and clinical outcomes.

## Background

Osseointegration refers to the direct structural and functional connection between living bone and the surface of a dental implant, ensuring long-term implant stability under functional loading [1]. The establishment of osseointegration occurs through a sequence of biological events including blood clot formation, inflammatory cell recruitment, angiogenesis and new bone formation at the implant surface. Osseointegration is not only a mechanical attachment, but also a biological process that involves different cells and healing events at the bone-implant interface [2]. Following this definition, understanding the biological events occurring during the early healing phase is essential.

**Fig. 1:**
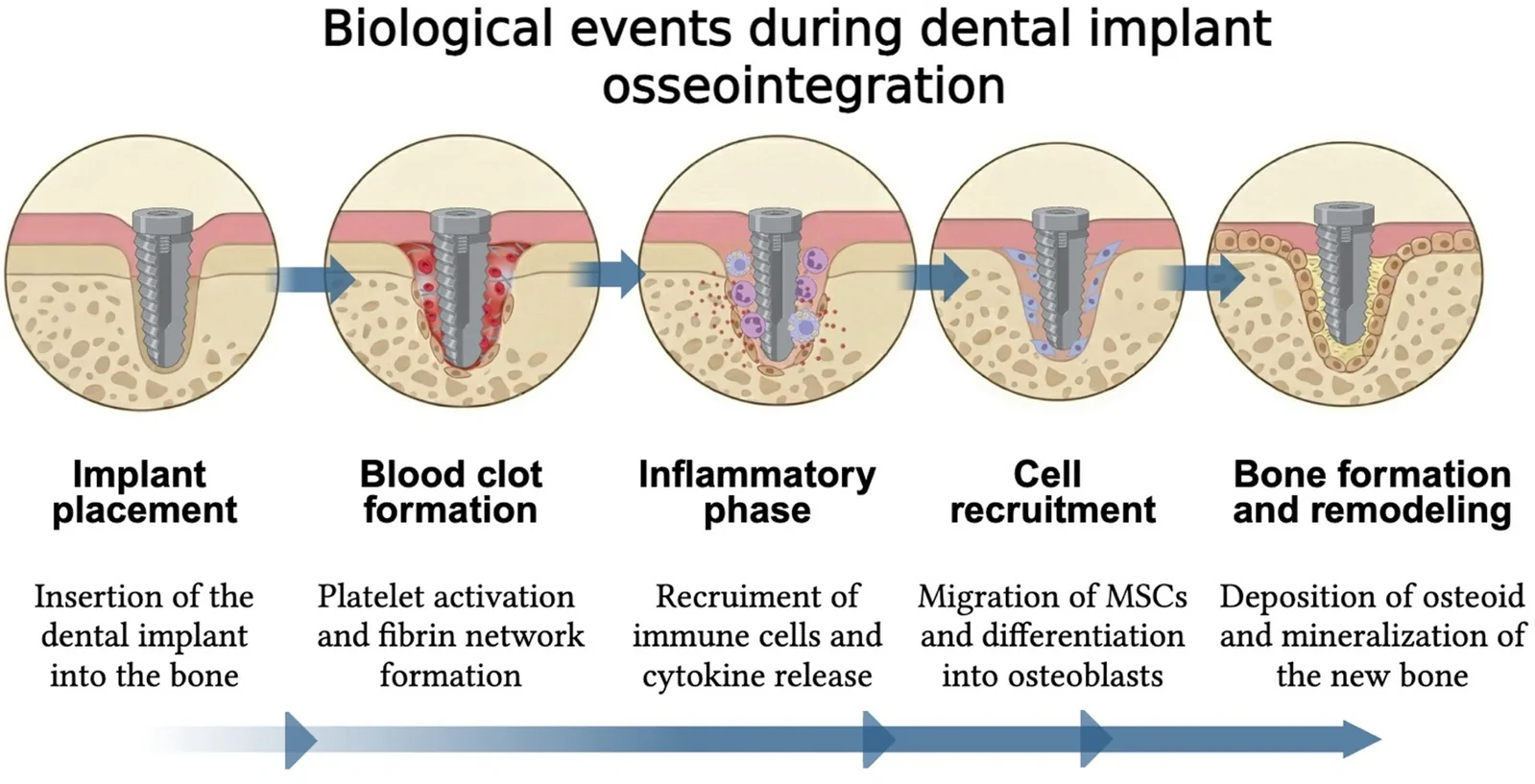
Schematic representation of the biological events of osseointegration following dental implant placement. The diagram illustrates blood clot formation, inflammatory response, cell recruitment, and new bone formation at the implant surface. Created in BioRender. Guel, D. (2026) https://BioRender.com/aisd3jt

The early healing phase is very important for the success of dental implants. Right after the implant is placed, a blood clot forms around the implant surface. This is followed by an inflammatory response, which represents a natural and necessary part of the healing process. During this phase, immune cells and bone-related cells are activated and start to prepare the area for new bone formation. If this early response is not properly regulated, the stability of the implant may be affected. In addition, the characteristics of the implant surface can influence how cells attach and respond during this initial period, which can further impact the integration process [2, 3]. These biological events are illustrated in Fig. 1.

**Fig. 2:**
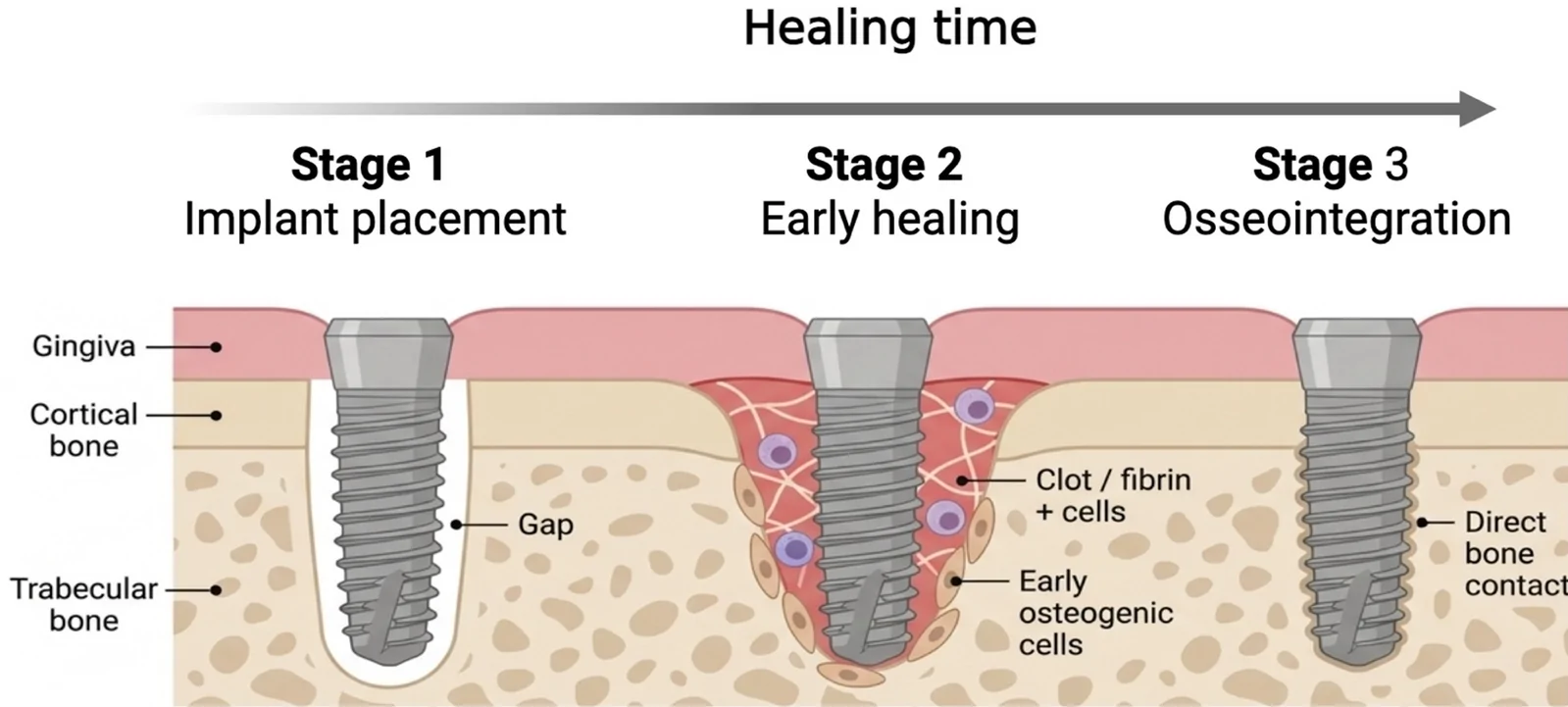
Healing timeline during dental implant osseointegration. The diagram shows the progression from implant placement to early healing and direct bone contact at the bone–implant interface. Created in BioRender. Guel, D. (2026) https://BioRender.com/wvqoume

The bone-implant interface is the area where the implant comes into direct contact with the surrounding bone tissue. This region is biologically active and involves different types of cells that respond to the implant surface. Osteoblasts play a key role in this process, as they are responsible for producing new bone matrix and contributing to bone formation around the implant [3]. The characteristics of the implant surface, such as its roughness and chemical composition, can influence how these cells attach, spread, and differentiate [3]. In addition, immune cells, particularly macrophages, contribute to the regulation of the local inflammatory response and may further affect the quality of osseointegration [4]. The progression of healing at the bone–implant interface is illustrated in Fig. 2.

**Fig. 3:**
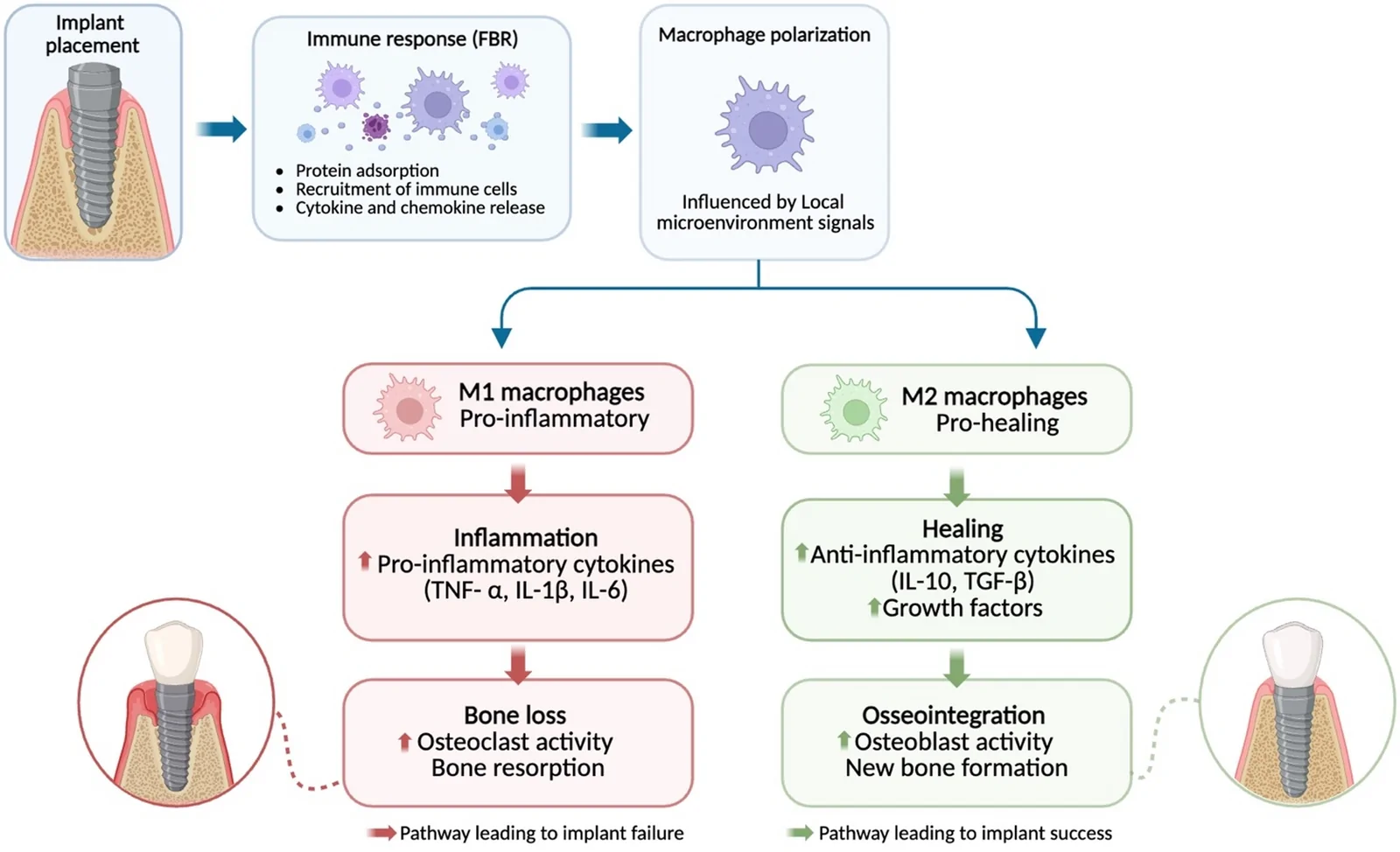
Immune-mediated mechanisms during implant osseointegration. Implant placement triggers a foreign body reaction (FBR), followed by macrophage activation. The M1/M2 model is shown as a simplified way to describe macrophage responses. Early inflammation is part of normal healing, while a shift toward a more pro-healing and regulatory response can support tissue repair and bone formation. Persistent or uncontrolled inflammation may contribute to bone loss. Created in BioRender. Guel, D. (2026) https://BioRender.com/otcff7t

Previous evidence indicates that the immune response plays a pivotal role in regulating bone formation during implant osseointegration, as it both promotes and constrains osteoblast activity through tightly controlled inflammatory processes [4]. Osseointegration is increasingly understood as an immune-regulated process rather than a passive bone response. The interaction between biomaterials and host tissues induces a foreign body reaction (FBR), which can either support integration or lead to implant failure depending on the balance of the immune response [5]. Controlled activation of macrophages promotes tissue healing and bone formation, whereas persistent inflammation and the formation of foreign body giant cells are associated with bone resorption and peri-implant disease [5]. Building upon this concept, macrophages have emerged as key regulators of the immune response during bone regeneration and osseointegration [6]. They orchestrate the balance between inflammation and tissue repair through their activation state. Although macrophage behaviour is often described using the simplified M1/M2 polarization model, macrophage phenotypes are dynamic and exist along a spectrum rather than as fixed states. In general, a predominantly pro-inflammatory response is important during the early healing phase, while a more pro-healing and regulatory response supports tissue repair, angiogenesis, and osteogenesis. Disruption of this balance may impair regeneration and contribute to fibrous encapsulation or bone loss [6]. This simplified concept of macrophage polarization in implant osseointegration is summarised in Fig. 3.

In addition to local biological processes, systemic factors may also influence bone healing and implant integration. From a clinical perspective, adequate vitamin D levels are essential for maintaining calcium and phosphate homeostasis and supporting normal bone metabolism [6]. Vitamin D deficiency has been associated with impaired bone remodelling and delayed skeletal healing and, in severe cases, may lead to rickets in children and osteomalacia, as well as contribute to osteoporosis in adults [6]. These conditions reflect defective mineralisation and reduced bone strength, which may ultimately compromise bone regeneration processes around implants.

Therefore, this narrative review aims to summarise current evidence on osteoblast responses to commonly used dental implant materials and to discuss the potential role of vitamin D in modulating biological processes involved in dental implant osseointegration.

## Literature search approach

A narrative literature review was performed to identify relevant evidence on dental implant materials, implant surface characteristics, osteoblast responses, vitamin D biology and dental implant osseointegration. The literature was identified using PubMed/MEDLINE, Google Scholar, and the reference lists of relevant articles and reviews. The literature was last updated in September 2026 before resubmission.

The main search concepts included dental implants, osseointegration, osteoblasts, titanium, zirconia, titanium-zirconium, Roxolid, implant surface, surface roughness, surface chemistry, coatings, vitamin D, vitamin D receptor, mineralisation, bone metabolism, early implant failure, and peri-implant bone healing. No formal date restriction was applied. Priority was given to systematic reviews, clinical studies, experimental in vitro and in vivo studies, and the relevant guidelines. This article was designed as a narrative review and not as a systematic review. Therefore, no PRISMA flow diagram, formal screening process, meta-analysis, or risk-of-bias assessment was performed.

The selected literature was critically synthesised to integrate the influence of implant materials and surface characteristics on osteoblast behaviour, the biological role of vitamin D in bone metabolism, and the currently limited evidence directly evaluating vitamin D in relation to specific implant materials or implant surfaces.

## Implant materials used in dental implants

### Titanium implants

Titanium dental implants show very high long-term survival rates and are considered a predictable treatment option in clinical practice. Systematic reviews with follow-up periods of more than 10 years report survival rates around 94–96%, with relatively limited marginal bone loss over time [7]. Nevertheless, implant survival does not necessarily equate to biological health of the peri-implant tissues.

Biological complications, such as peri-implant mucositis and peri-implantitis, may occur during long-term follow-up. Supportive periodontal care seems to play an important role in maintaining implant stability and reducing disease progression [8].

In addition, patient-related factors, including systemic diseases and the use of antiresorptive medications, have been associated with altered implant outcomes and should be carefully considered during treatment planning. According to ITI consensus reports, thorough case selection and comprehensive risk assessment are essential for achieving long-term implant success [9].

Despite the excellent clinical performance of titanium implants, aesthetic limitations, as well as patient perceptions regarding metal-based materials, have encouraged the investigation of alternative implant materials. Furthermore, the selection of implant materials is not solely based on material properties but is also influenced by patient-related factors, including individual preferences, material availability, and the need for a personalised treatment approach [9].

### Zirconia implants

Zirconia dental implants have gained increasing attention due to their high biocompatibility and favourable soft tissue response. Clinical studies and systematic reviews have reported promising osseointegration and biological performance of zirconia implants [10].

At the bone-implant interface, the biological response to zirconia appears to be strongly influenced by surface properties, including roughness, topography, and surface chemistry. In vitro evidence indicates that osteoblast adhesion and subsequent differentiation on zirconia surfaces are regulated by integrin-related mechanisms and heparin-sensitive molecules [11]. Modified zirconia surfaces have also been shown to support osteoprogenitor cell attachment, spreading, and migration, with cellular responses comparable to those observed on titanium surfaces [12]. In addition, hierarchically roughened zirconia surfaces have demonstrated enhanced osteogenic differentiation and improved osseointegration compared with machined/smoother zirconia surfaces, suggesting that surface design plays an important role in the bone response to zirconia implants [13]. Recent reviews report that peri-implant tissues surrounding zirconia components exhibit comparable or reduced inflammatory response compared with titanium, with lower bleeding on probing and decreased plaque accumulation in several clinical observations [14, 15].

Histological evidence indicates that peri-implant mucosal dimensions, epithelial attachment, and connective tissue organisation around zirconia are comparable to titanium, supporting adequate biological integration and stability of the soft-tissue seal [15]. At the cellular level, zirconia surfaces promote favourable human gingival fibroblast adhesion and proliferation, demonstrating comparable and, in some cases, superior cellular responses compared with titanium surfaces, supporting their suitability as implant and abutment materials [16].

From an aesthetic perspective, zirconia offers clear advantages due to its tooth-coloured appearance, reducing the risk of mucosal discolouration, particularly in thin biotype patients and in the anterior region [14]. It is also important to distinguish between different zirconia implant designs. One-piece and two-piece zirconia implants may differ in their prosthetic flexibility, technical behaviour, and clinical indications. Therefore, biological outcomes related to soft-tissue response or osseointegration should be interpreted separately from technical outcomes such as prosthetic complications, fracture risk, or limitations in prosthetic restoration [9, 10, 17].

### Titanium-zirconium (Ti-Zr) alloys

**Fig. 4:**
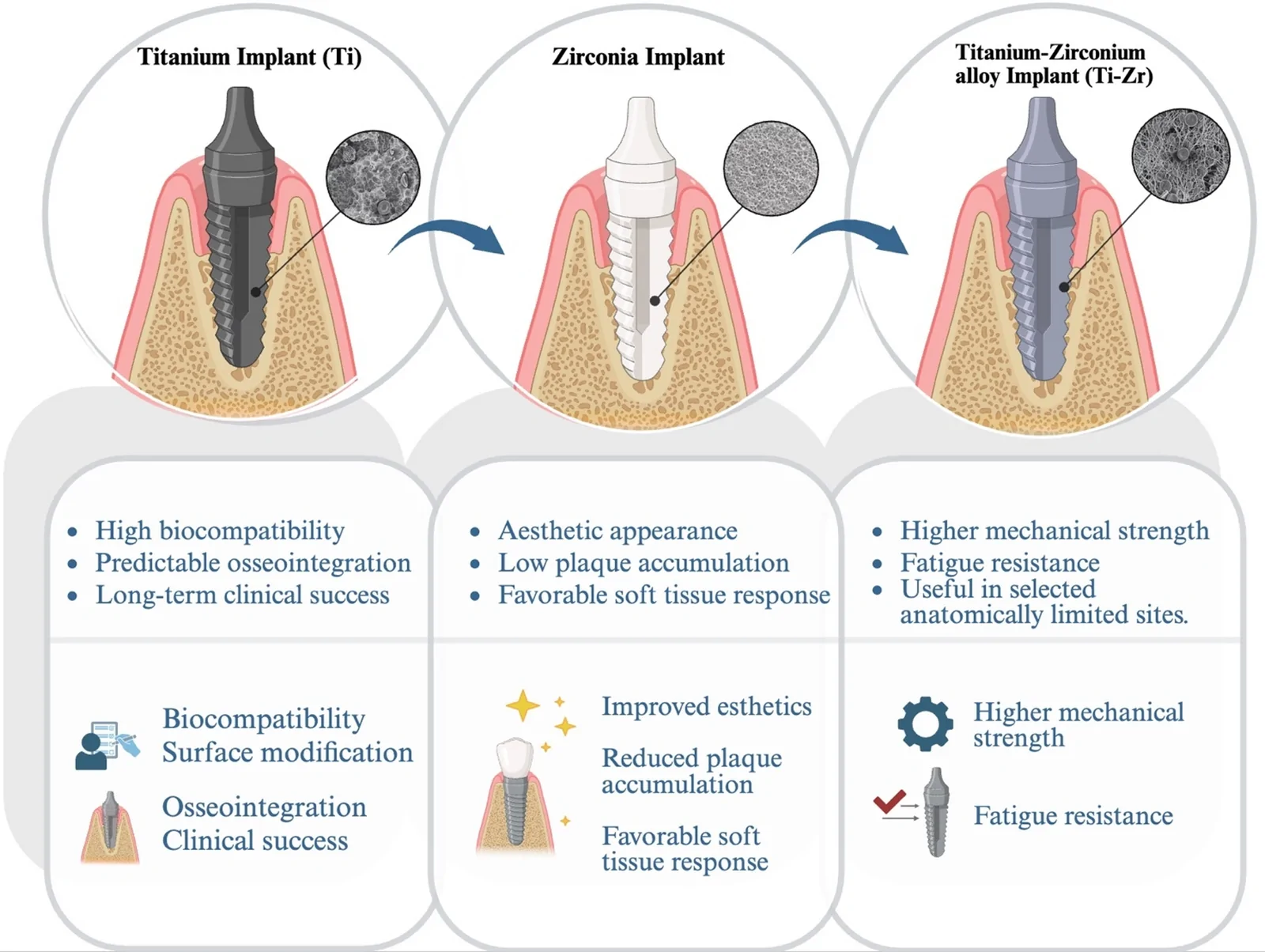
Comparison of commonly used dental implant materials. Schematic illustration comparing commercially pure titanium, zirconia, and titanium-zirconium alloy implants. Titanium implants are widely used due to their established biocompatibility and predictable osseointegration. Zirconia implants offer aesthetic advantages and favourable soft-tissue responses, although clinical outcomes may depend on implant design, surface characteristics, and prosthetic configuration. Titanium-zirconium alloys provide enhanced mechanical strength and fatigue resistance, supporting their use in selected reduced-diameter implants and anatomically limited sites. Created in BioRender. Guel, D. (2026) https://BioRender.com/ox0y3wa

Titanium-zirconium (Ti-Zr) alloys were developed to address the mechanical limitations of commercially pure titanium, particularly in narrow-diameter implants and high-load clinical situations [18]. Although titanium demonstrates excellent biocompatibility and predictable osseointegration, its mechanical resistance may be insufficient in reduced bone volume or high-stress areas. The incorporation of zirconium (typically 13–15 wt%) into titanium has been reported to significantly enhance tensile and fatigue strength while maintaining corrosion resistance and biocompatibility [18]. This improved mechanical performance enables the use of smaller-diameter implants with acceptable fracture resistance, offering clinical advantages in anatomically limited sites [18]. Additionally, Ti-Zr alloys provide high strength without the inclusion of aluminium and vanadium found in Titanium-6 aluminium-4 vanadium (Ti-6Al-4 V) alloys, elements that have raised biocompatibility concerns [18]. Preclinical and experimental evidence indicates that Ti-Zr alloys demonstrate comparable bone-to-implant contact and osseointegration patterns to commercially pure titanium, supporting their biological reliability in vivo. Clinical evidence further supports the use of Ti-Zr implants. A systematic review of randomized controlled trials reported comparable or improved survival rates and marginal bone level stability for titanium-zirconium implants compared with commercially pure titanium implants [17]. A comparative overview of the three implant materials is provided in Fig. 4.

## Osteoblast responses to implant surfaces

### Osteoblast adhesion and early cellular interactions

Osteoblasts are the main bone-forming cells and play a central role in osseointegration [19]. They are responsible for producing bone matrix and regulating its mineralisation around the implant surface [20]. During the early healing phase, osteoblasts attach to the implant surface through interactions mediated by adsorbed proteins and integrin receptors, forming focal adhesions that influence cytoskeletal organisation and intracellular signalling pathways [19, 20]. Proper cell adhesion and spreading are essential, as they determine subsequent cellular behaviour and the capacity for bone formation [20].

**Fig. 5:**
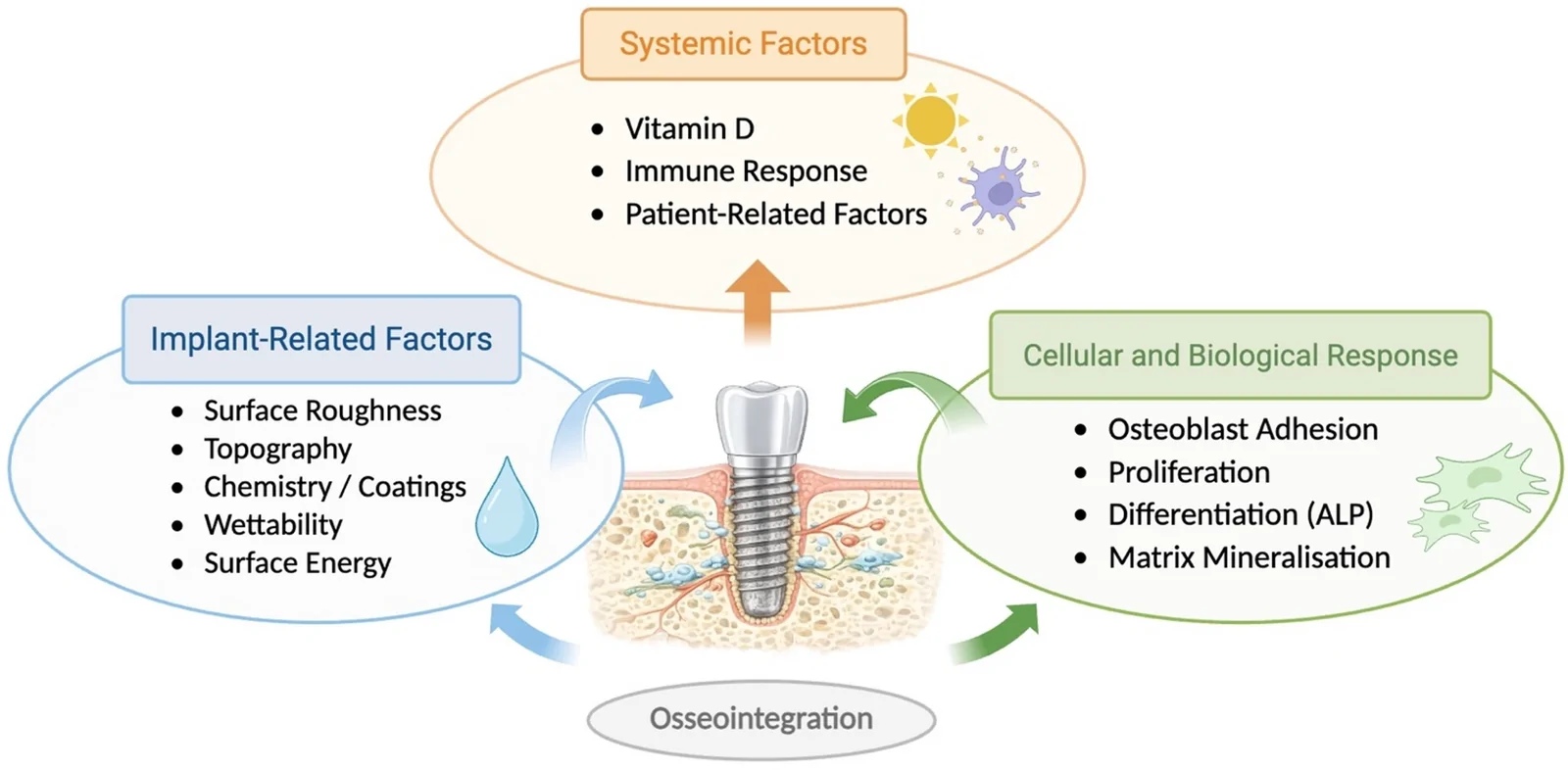
Main factors affecting osteoblast behaviour and osseointegration. Implant-related surface properties, systemic factors, and cellular responses interact to influence osteoblast adhesion, proliferation, differentiation, and bone formation around the implant. Created in BioRender. Guel, D. (2026) https://BioRender.com/81evmyc

As shown in Fig. 5, both implant surface properties and systemic factors, including vitamin D, contribute to the regulation of osteoblast behaviour and the overall process of osseointegration.

Following initial adhesion, osteoblasts subsequently proliferate and differentiate. Changes in osteoblast morphology are closely related to their functional activity. On more favourable implant surfaces, osteoblasts typically exhibit a well-spread and flattened morphology with organised actin cytoskeleton, whereas less favourable surfaces may result in a more rounded cell shape. These morphological characteristics are considered indicators of cytoskeletal stability and are associated with enhanced osteogenic differentiation and matrix production [19, 20]. Surface topography and micro-scale roughness have been shown to modulate osteoblast proliferation and enhance the expression of osteogenic markers such as alkaline phosphatase and collagen type I [19, 21, 22]. In addition, bioactive surface modifications, including nanohydroxyapatite coatings, further stimulate osteoblast differentiation and promote early mineralised matrix formation, thereby supporting more rapid bone integration [21]. Collectively, these biological processes demonstrate that osteoblast adhesion, proliferation, and early differentiation are closely influenced by implant surface characteristics [19, 21].

### Implant surface properties influencing osteoblast behaviour

Implant surface characteristics significantly influence osteoblast behaviour and the overall process of osseointegration. Micro- and nano-scale surface roughness has been reported to enhance osteoblast adhesion and spreading compared to smoother surfaces [19, 23], while moderately rough topographies promote increased osteogenic differentiation and extracellular matrix production [19, 22]. These surface modifications improve mechanical interlocking with bone and support early cellular attachment [23].

In addition to roughness, surface functionalisation strategies play an important role in modulating cellular responses. Bioactive coatings, such as nanohydroxyapatite layers, have been reported to enhance osteoblast proliferation, increase alkaline phosphatase activity, and stimulate mineralised matrix formation, thereby accelerating early bone formation [21]. Such coatings aim to create a more biomimetic surface that resembles the mineral phase of bone.

**Fig. 6:**
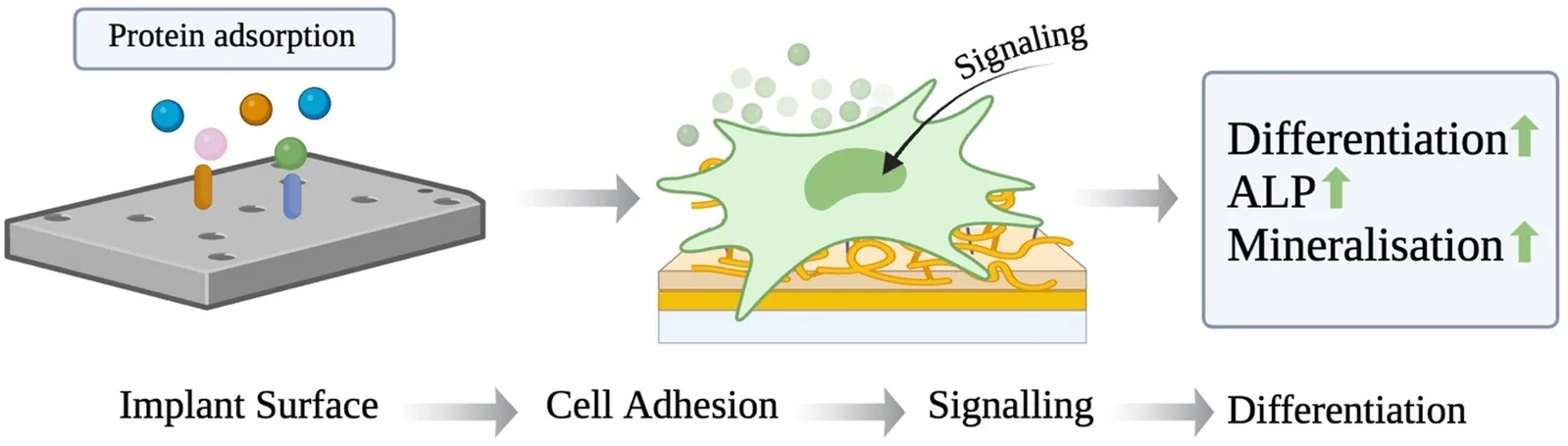
Osteoblast interactions with implant surfaces. Protein adsorption onto the implant surface facilitates cell adhesion and activates intracellular signalling pathways that regulate gene expression, osteogenic differentiation, and mineralisation. Created in BioRender. Guel, D. (2026) https://BioRender.com/8ac13yw

Furthermore, implant surface properties can influence the local immune environment. Surface characteristics that promote a pro-healing macrophage phenotype may indirectly enhance osteogenesis and improve the quality of bone regeneration around implants [4]. Collectively, these biological processes demonstrate that osteoblast adhesion, proliferation, and early differentiation are closely influenced by implant surface characteristics, as illustrated in Fig. 6.

## Experimental approaches to evaluate osteoblast responses

### Adhesion and morphology

In vitro studies that evaluate dental implant materials mainly focus on specific cellular outcomes related to osteoblast behaviour. There is no single “gold standard” assay that can fully assess the osteogenic potential of an implant surface [24]. Instead, a combination of different assays is required to obtain a more complete understanding of how cells respond to a material [24]. The most studied parameters include cell adhesion, morphology, proliferation and osteogenic differentiation.

Cell attachment to a surface is considered a necessary first step for subsequent cell survival, proliferation and differentiation [24]. The formation of focal adhesion points and cytoskeletal organisation plays an important role in regulating osteoblast behaviour [3, 24]. Surface topography and roughness can significantly influence cell spreading and attachment, as demonstrated in several in vitro studies evaluating modified titanium surfaces [25]. Adhesion and morphology are typically assessed using microscopy techniques and cytoskeletal staining [24].

### Proliferation assays

Cell proliferation is commonly assessed indirectly using metabolic or viability assays, such as MTT, Alamar Blue/resazurin, or CellTiter-Glo (CTG). These assays provide useful information about cellular metabolic activity and viable cell number over time. However, they should not be interpreted as direct proliferation assays unless supported by additional methods. Therefore, results from these assays should be described carefully and interpreted together with other markers of cell behaviour and osteogenic differentiation [24].

There is a reciprocal relationship between proliferation and differentiation, as osteoblastic cells usually undergo an initial proliferative phase before entering a differentiation phase [24]. Therefore, proliferation assays provide useful information about cell growth on implant surfaces but should always be interpreted together with markers of osteogenic differentiation.

Mesenchymal stem cells (MSCs) undergo significant metabolic changes during osteogenic differentiation. Specifically, osteogenic differentiation is associated with a metabolic shift from glycolysis toward increased mitochondrial oxidative phosphorylation [26]. Beyond energy production, mitochondrial activity has been shown to regulate key signalling pathways involved in osteogenesis, such as β-catenin activation [27]. This indicates that mitochondrial function plays an active regulatory role in directing stem cell fate toward osteogenic lineage commitment. Furthermore, external factors such as extracellular matrix stiffness have been reported to influence cellular metabolism, thereby promoting osteogenic differentiation [28]. These findings highlight the critical interplay between cellular metabolism and the microenvironment in regulating osteogenesis. Importantly, this interplay is directly relevant to biomaterial surfaces used in implant dentistry, as surface properties can modulate cellular metabolic activity and, consequently, influence osteogenic outcomes.

### Osteogenic differentiation (ALP)

**Fig. 7:**
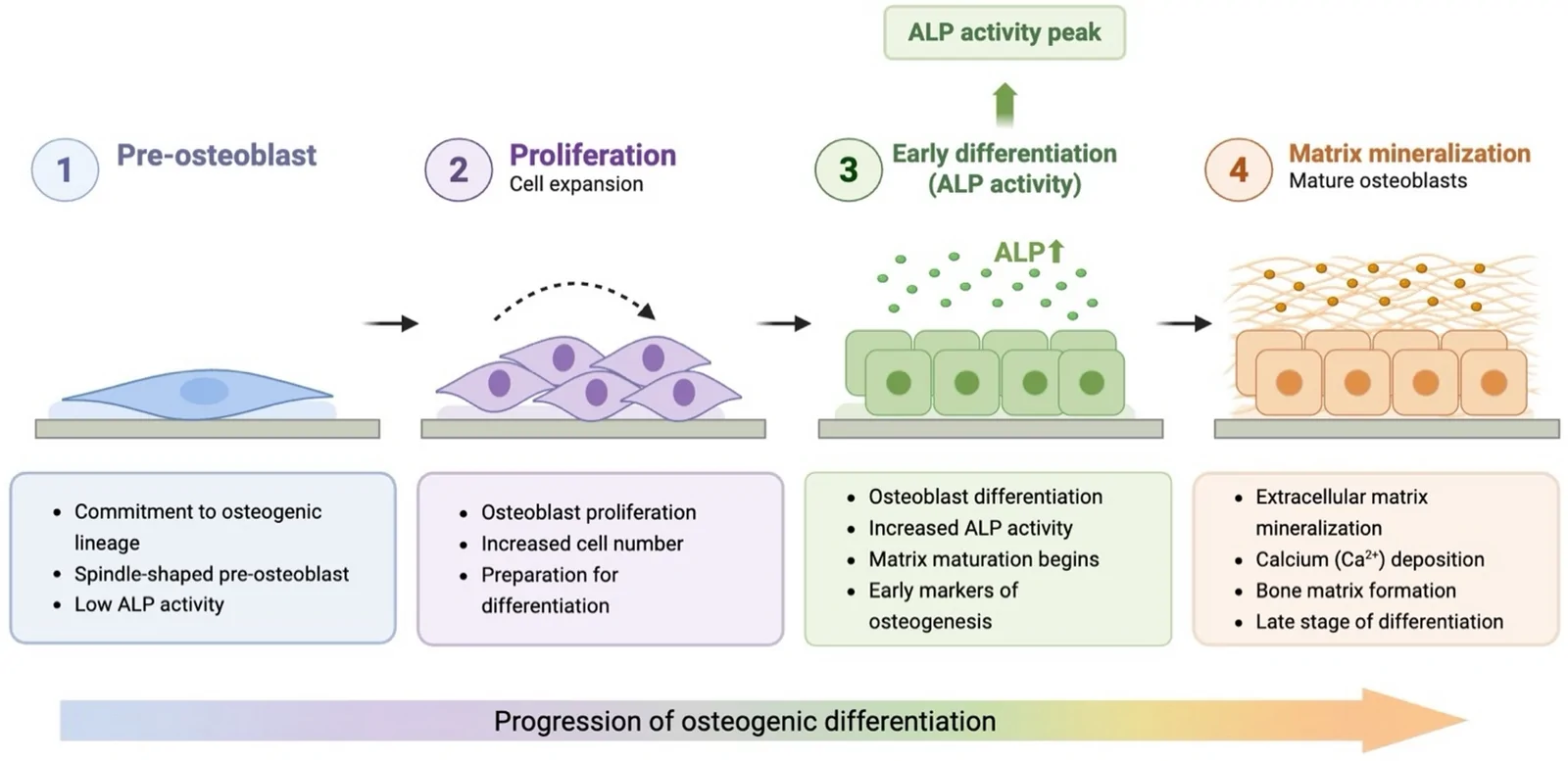
Osteogenic differentiation and alkaline phosphatase activity. Sequential stages of osteoblast differentiation are shown, with increased alkaline phosphatase (ALP) activity during early differentiation and subsequent matrix mineralisation. Created in BioRender. Guel, D. (2026) https://BioRender.com/fiigxd1

Alkaline phosphatase (ALP) activity is widely used as an early marker of osteogenic differentiation, as illustrated in Fig. 7 [24, 29]. However, ALP activity is time- and model-dependent, and increased ALP alone does not confirm complete mineralisation or improved clinical osseointegration.

ALP is involved in early matrix maturation and contributes to the initiation of mineralisation by increasing the local availability of inorganic phosphate [24]. Several in vitro studies have shown that surface modifications of titanium implants can influence ALP activity levels [25]. Late-stage osteogenic differentiation is commonly assessed by evaluating mineralised matrix deposition, typically using Alizarin Red S staining to detect calcium deposits.

ALP expression is closely linked to cellular metabolic activity during osteogenic differentiation. Mesenchymal stem cells undergo a metabolic shift from glycolysis to increased mitochondrial oxidative phosphorylation, which supports the elevated energy demands of osteoblast differentiation [26]. This metabolic reprogramming supports osteogenic differentiation and has been associated with increased expression of osteogenic markers, including ALP.

In addition, mitochondrial activity can regulate key osteogenic signalling pathways, including β-catenin signalling, thereby further promoting ALP expression and early differentiation processes [27]. Therefore, ALP activity is widely used as a key early indicator to evaluate the osteogenic potential of implant surfaces, as biomaterial properties such as surface roughness, chemistry, and topography can directly influence cellular metabolism and differentiation outcomes [25].

### Mineralisation assays

Late-stage osteogenic differentiation is commonly assessed by evaluating mineralised matrix deposition. Alizarin Red S staining is widely used to detect calcium deposits and mineralised nodules formed by differentiating osteoblasts [24, 29]. Increased mineral deposition has been reported on certain modified implant surfaces in vitro [25].

Although in vitro studies provide valuable insights, their interpretation requires caution. Results may vary depending on cell source, passage number, seeding density and culture conditions [24]. In addition, in vitro models cannot fully replicate the complex biological environment of bone healing in vivo [24]. Therefore, findings from in vitro assays cannot be directly translated into clinical outcomes without further validation.

## Vitamin D biology

### Vitamin D metabolism and activation

**Fig. 8:**
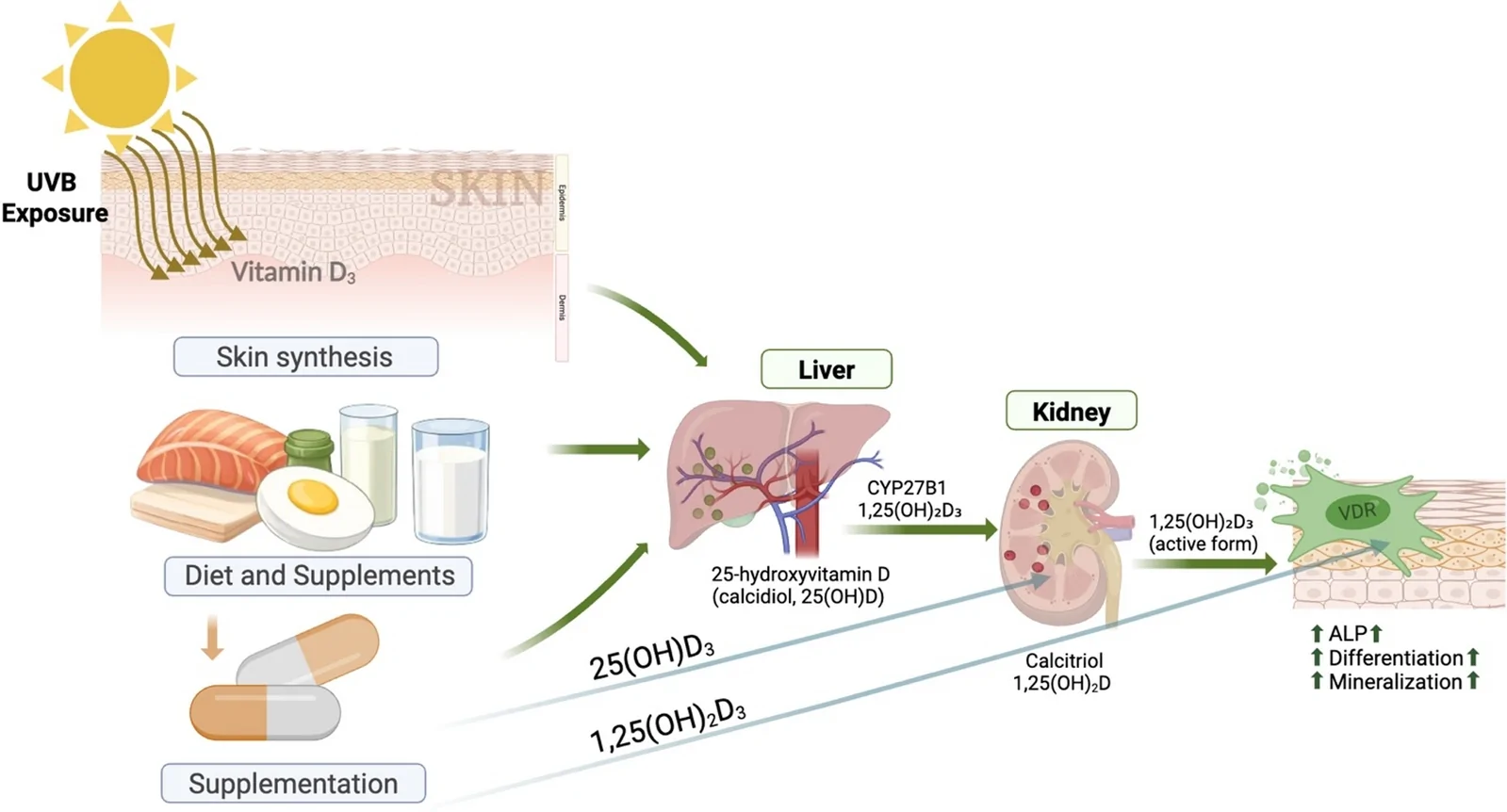
Overview of vitamin D metabolism. Vitamin D is produced in the skin after UVB exposure and obtained from diet and supplementation. It is converted in the liver to 25-hydroxyvitamin D [25(OH)D] and in the kidney to its active form, 1α,25-dihydroxyvitamin D₃ [1α,25(OH)₂D₃], which acts on target cells via the vitamin D receptor (VDR). Created in BioRender. Guel, D. (2026) https://BioRender.com/yioqy89

Vitamin D is a fat-soluble vitamin that requires metabolic activation to exert its biological effects. After its synthesis in the skin through ultraviolet B (UVB) exposure or its intake through diet, vitamin D is transported in the bloodstream bound to vitamin D-binding protein (DBP) [30]. The first activation step occurs in the liver, where vitamin D undergoes 25-hydroxylation to form 25-hydroxyvitamin D [25(OH)D], which represents the main circulating form used to assess vitamin D status. A second hydroxylation step occurs primarily in the kidney, where the enzyme 1α-hydroxylase converts 25(OH)D into 1α,25-dihydroxyvitamin D₃ [1α,25(OH)₂D₃], the biologically active form of vitamin D. This active metabolite binds to the vitamin D receptor (VDR) and regulates the expression of genes involved in calcium homeostasis and bone metabolism [30]. These sequential steps in vitamin D metabolism are summarised in Fig. 8.

### Sources of vitamin D

Vitamin D can be obtained from two main sources: endogenous synthesis in the skin and dietary intake. Cutaneous production occurs when UVB radiation converts 7-dehydrocholesterol in the skin into vitamin D₃. Dietary sources of vitamin D include fatty fish, egg yolk, fortified dairy products, and nutritional supplements [31].

### Prevalence of vitamin D deficiency

Vitamin D deficiency is considered a global public health concern affecting populations worldwide. Several factors contribute to low vitamin D levels, including limited sunlight exposure, skin pigmentation, ageing, obesity, and insufficient dietary intake. In addition, modern lifestyle patterns and reduced outdoor activity have been associated with increasing prevalence of vitamin D deficiency across different age groups [32].

### Vitamin D deficiency in Europe

Populations living in Europe often present suboptimal vitamin D levels during large parts of the year, especially in regions with reduced sunlight exposure during winter months. This is largely attributed to the higher latitudes of many European countries, where UVB radiation is insufficient for cutaneous vitamin D synthesis during extended periods of the year. In addition, seasonal variation, limited outdoor activity, and lifestyle factors further contribute to reduced vitamin D production in these populations [32, 33]. These findings highlight the widespread occurrence of vitamin D deficiency and emphasize the importance of understanding its biological effects, particularly in tissues involved in bone metabolism. Vitamin D plays a fundamental role in bone metabolism by regulating calcium and phosphate homeostasis and influencing osteoblast and osteoclast activity [6].

### Clinical relevance

Vitamin D plays an essential role in skeletal health by regulating calcium and phosphate homeostasis and supporting normal bone remodelling. Adequate vitamin D levels are necessary for proper osteoblast differentiation and mineralised matrix formation. Consequently, insufficient vitamin D status may negatively influence bone metabolism and potentially affect the biological processes involved in bone healing and implant osseointegration [30].

## Role of vitamin D in osteoblast function

In addition to material-related factors, osteoblast behaviour is also influenced by systemic and biochemical regulators that play a key role in bone metabolism. Among these factors, vitamin D has been widely studied due to its regulatory effects on osteoblast activity and bone formation [6]. Vitamin D plays an important role in regulating osteoblast function, particularly through its interaction with the VDR [34].

### Vitamin D receptor signalling in osteoblasts

Experimental studies have shown that osteoblasts express the VDR, which allows them to directly respond to vitamin D [34]. When osteoblasts are exposed to vitamin D, changes in the expression of genes related to differentiation and matrix formation can be observed [34]. The involvement of VDR in these effects has been confirmed in functional studies. In human osteoblast cultures, inhibition of the nuclear VDR significantly reduced the increase in ALP activity and mineralisation induced by vitamin D [35]. This suggests that the effects of vitamin D on osteoblast differentiation are mainly mediated through VDR-dependent mechanisms.

Further evidence indicates that vitamin D also regulates downstream transcriptional factors that are involved in mineralisation. For example, changes in Dickkopf-related protein 1 (DKK1), a known inhibitor of the Wnt/β-catenin signalling pathway, and CCAAT/enhancer-binding protein beta (C/EBPβ), a transcription factor involved in osteoblast differentiation, have been reported following vitamin D treatment, linking VDR activation to signalling pathways involved in matrix mineralisation [36]. Overall, available in vitro data support that vitamin D directly regulates osteoblast function through VDR-mediated signalling pathways, influencing gene expression, differentiation, and matrix mineralisation.

### Effects of vitamin D on osteoblast differentiation

Several studies have investigated how vitamin D affects osteoblast activity. One of the most consistent findings is the increase in ALP activity, which is considered an early marker of osteogenic differentiation [6]. Zarei et al. [37] showed that treatment of human bone marrow-derived stromal cells and murine osteoblasts with vitamin D led to a dose-dependent increase in ALP activity. Similar results were reported by van Driel et al. [35], who observed significant upregulation of ALP activity in human osteoblast cell lines after exposure to vitamin D. These findings indicate that vitamin D promotes early osteoblast differentiation in both human and animal cell models.

### Vitamin D and mineralised matrix formation

In addition to ALP activity, vitamin D has been shown to enhance mineralised matrix formation. Increased calcium deposition, evaluated using Alizarin Red staining, was reported in osteoblast cultures treated with vitamin D [35, 37]. The effects were generally dose-dependent, although very high concentrations did not always result in further stimulation, suggesting that the response may depend on the concentration used.

It is important to note that increased ALP activity alone does not automatically mean complete mineralisation. Jo et al. [36] demonstrated that inhibition of DKK1 reduced vitamin D-induced mineral deposition without significantly affecting ALP activity. This finding suggests that additional signalling mechanisms are required for full matrix mineralisation. Taken together, current in vitro evidence shows that vitamin D enhances osteoblast differentiation by increasing ALP activity and promoting mineralised matrix formation, mainly through VDR-related pathways [34]. These cellular effects suggest that vitamin D may influence the biological processes involved in bone healing and regeneration, which are essential for successful implant osseointegration.

## Implications for implant osseointegration

### Bone healing and osseointegration

Bone healing around dental implants is a complex biological process that involves inflammation, bone remodelling, and new bone formation at the bone-implant interface. Osseointegration is defined as the direct structural and functional connection between living bone and the surface of a load-bearing implant. Successful osseointegration depends mainly on the coordinated activity of osteoblasts, which are responsible for bone matrix formation and mineralisation, and osteoclasts, which regulate bone resorption and remodelling at the implant surface [3].

### Vitamin D and osteoblasts: mechanism

Since vitamin D plays a key role in regulating osteoblast differentiation, gene expression, and mineralised matrix formation, it may influence biological processes involved in implant integration [6, 34]. In vitro studies have shown that vitamin D enhances osteogenic activity through VDR-mediated pathways, leading to increased ALP activity and mineralised matrix formation in osteoblast cultures [35–37]. These cellular effects suggest that vitamin D may contribute to improved bone formation during the early stages of implant healing.

### Clinical relevance

From a clinical perspective, vitamin D status may be relevant to implant healing because vitamin D is involved in bone metabolism and osteoblast activity [6, 34]. However, biological relevance should be clearly distinguished from direct clinical evidence, because current human studies remain limited and heterogeneous [38, 39].

A retrospective clinical study reported a higher incidence of early dental implant failure in patients with lower serum vitamin D levels [40]. However, this difference was not statistically significant, and the group with severe vitamin D deficiency was small. Therefore, this finding should be interpreted as a non-significant trend rather than as a proven association. Recent systematic reviews also suggest a possible positive role of vitamin D in osseointegration, but the available human evidence remains inconsistent because of differences in study design, vitamin D thresholds, supplementation protocols, implant sites, follow-up periods, and outcome measures [38, 39].

An umbrella review also concluded that the available evidence should be interpreted with caution because of limitations in the existing systematic reviews [41]. A recent scoping review further showed that most clinical evidence on dietary factors and implant outcomes focuses on vitamin D, while evidence for other nutrients remains limited [42].

Current guideline-based recommendations do not support routine untargeted vitamin D testing or supplementation solely for dental implant treatment [43]. Nevertheless, individual assessment may be considered in selected clinical situations, such as suspected or known vitamin D deficiency, systemic factors affecting bone metabolism, or unexplained early implant failure [43].

Emerging clinical evidence on topical vitamin D application around immediate implants has shown promising short-term clinical and radiographic results [44]. However, this evidence should still be interpreted carefully, because the study was limited in sample size, follow-up duration, and outcome measures. Further well-designed prospective clinical studies are needed before firm conclusions can be drawn regarding the effect of vitamin D on implant survival or osseointegration.

### Implant materials and vitamin D

The interaction between vitamin D and implant materials should be interpreted through the biological response to the implant surface. Surface characteristics can influence osteoblast adhesion, differentiation, and mineralised matrix formation [3, 23]. Vitamin D is also involved in bone metabolism and osteoblast activity [6, 34].

However, direct evidence showing whether vitamin D-related effects differ between titanium, zirconia, and titanium-zirconium implant surfaces remains limited [38, 39]. Therefore, differences in osteoblast response should not be attributed only to the bulk material, because surface roughness, topography, chemistry, and experimental conditions may also influence the biological outcome.

#### Titanium and vitamin D

Titanium implant surfaces have been widely shown to support osteoblast adhesion, proliferation, and differentiation. In vitro studies indicate that surface characteristics, such as roughness and topography, can modulate osteogenic activity, including ALP expression and mineralised matrix formation, reflecting enhanced osteogenic potential [23, 45]. Since vitamin D is involved in osteoblast differentiation and mineralisation through VDR-mediated pathways, it may also influence osteogenic responses on titanium surfaces [6, 34]. However, direct evidence specifically evaluating vitamin D-related effects on titanium implant surfaces remains limited [38, 39].

#### Zirconia and vitamin D

Similarly, zirconia implant surfaces have been shown to support osteoblast adhesion, proliferation, and differentiation, with cellular responses regulated by surface properties and integrin-mediated mechanisms [11]. In vitro studies further indicate that zirconia surfaces can promote osteogenic cell behaviour comparable to that observed on titanium surfaces [12]. Surface characteristics such as roughness and topography play an important role in modulating osteoblast adhesion, differentiation, and osseointegration on zirconia surfaces [13]. However, direct evidence specifically investigating the interaction between vitamin D and zirconia implant surfaces remains limited [38, 39]. Therefore, vitamin D-related effects around zirconia implants should currently be interpreted mainly based on general vitamin D and bone metabolism evidence rather than on zirconia-specific clinical studies.

#### Titanium-zirconium alloys and vitamin D

Titanium-zirconium alloys have been introduced as an alternative to commercially pure titanium, combining improved mechanical strength with favourable biological and clinical performance. Evidence from systematic reviews indicates that Ti-Zr implants demonstrate survival and success rates comparable to titanium implants, with similar clinical outcomes, particularly in reduced-diameter applications [17, 18].

To our knowledge, no studies identified in the present narrative review have specifically investigated the interaction between vitamin D and titanium-zirconium implant surfaces. Therefore, it remains unclear whether vitamin D status may influence the biological response to Ti-Zr implants differently compared with titanium or zirconia implants [38, 39]. Further in vitro, in vivo, and clinical studies are needed to clarify this possible interaction.

### Integrated effects of implant materials and vitamin D on osseointegration

Overall, the available literature shows that implant οsseointegration is influenced by both material-related and host-related factors. Implant surface characteristics, including roughness, topography, chemistry, and hydrophilicity, can influence osteoblast adhesion, differentiation, and mineralised matrix formation [3, 23]. Titanium remains the most established implant material, while zirconia and titanium-zirconium alloys have shown favourable biological and clinical performance in selected indications [7, 10, 17, 18].

At the same time, vitamin D is biologically relevant for bone metabolism, osteoblast activity, and mineralisation [6, 34]. Recent systematic reviews suggest a possible relationship between vitamin D status and implant outcomes, but the available human evidence remains limited and heterogeneous [38, 39, 41, 46]. In addition, a retrospective clinical study reported an increase incidence of early implant failure in patients with lower serum vitamin D levels, although this finding was not statistically significant [40]. Therefore, vitamin D deficiency should currently be considered as a possible modifying factor rather than as an isolated or proven cause of implant failure.

For example, local delivery of bioactive agents and chronic inflammatory conditions may influence osseointegration, peri-implant tissue responses, and marginal bone stability [47, 48]. This supports the concept that implant outcomes are not determined by the implant material alone, but by the interaction between material properties, local biological conditions, and systemic host-related factors.

Importantly, most available studies evaluate vitamin D in relation to bone healing or implant outcomes in general, without clearly distinguishing between titanium, zirconia, and titanium-zirconium implants. As a result, it remains unclear whether vitamin D status interacts differently with specific implant materials or surface characteristics. Future in vitro, in vivo, and clinical studies should therefore assess vitamin D-related effects together with clearly defined implant materials, surface properties, and clinical outcomes. The key evidence directly evaluating or summarising the role of vitamin D in relation to dental implant osseointegration is summarised in Table 1.

**Table 1:** Summary of key evidence on vitamin D and dental implant osseointegration

Study	Design / model	Implant / vitamin D context	Main message	Key limitation
Werny et al., 2022 [38]	Systematic review	Animal and human studies, vitamin D deficiency/supplementation	Animal data suggest possible effects on osseointegration, human evidence limited	Few human studies and heterogeneous evidence
Buzatu et al., 2024 [39]	Human systematic review	Serum vitamin D and implant outcomes	Possible relationship between vitamin D status and implant outcomes	Few studies and heterogeneous designs/outcomes
Miron et al., 2025 [46]	Systematic review	Vitamin D deficiency, osseointegration, early implant failure	Available studies suggest a possible positive association with adequate vitamin D	Not material-specific, heterogeneous evidence
Mangano et al., 2018 [40]	Retrospective clinical study	Serum vitamin D categories and early implant failure	Numerical increase in early failure with lower vitamin D	Not statistically significant, with a small severe-deficiency group
Tallon et al., 2024 [41]	Umbrella review	Vitamin D, bone metabolism, implant outcomes	Possible positive role of vitamin D	Based on reviews with limitations
Sodnom-Ish et al., 2026 [42]	Scoping review	Nutritional factors, mainly vitamin D	Most nutrition-related implant evidence focuses on vitamin D	Broad scope and heterogeneous evidence
DGI/DGZMK, 2025 [43]	S3 guideline	Vitamin D testing/supplementation	Routine untargeted testing/supplementation solely for implants not recommended	Individual assessment still relevant in selected cases
Ayyad et al., 2025 [44]	Randomized clinical trial	Topical vitamin D around immediate implants	Promising short-term clinical/radiographic findings	Small sample, short follow-up, and indirect outcome measures

## Conclusions

Dental implant osseointegration depends on a complex interaction between implant material characteristics, surface properties, osteoblast responses, and host-related biological factors. Surface roughness, topography, chemistry, and other surface-related features can influence osteoblast adhesion, differentiation, and mineralised matrix formation. Therefore, biological differences between titanium, zirconia, and titanium-zirconium implants should not be attributed only to the bulk material itself.

Vitamin D is biologically relevant for bone metabolism and osteoblast activity, and experimental evidence suggests that it may support osteogenic differentiation and mineralised matrix formation. However, current human clinical evidence remains limited and heterogeneous. At present, there is insufficient evidence to show that vitamin D has different effects on osseointegration around titanium, zirconia, or titanium-zirconium implants. Future in vitro, in vivo, and clinical studies should evaluate vitamin D-related effects together with clearly defined implant materials, surface characteristics, and clinical outcomes. This may help clarify whether vitamin D status or supplementation has specific importance for different implant materials in dental implant osseointegration.

## Data Availability

No datasets were generated or analysed during the current study.

## Abbreviations

ALP: Alkaline phosphatase
C/EBPβ: CCAAT/enhancer-binding protein beta
CTG: CellTiter-Glo
DKK1: Dickkopf-related protein 1
DBP: Vitamin D-binding protein
FBR: Foreign body reaction
M1: Classically activated macrophage phenotype
M2: Alternatively activated macrophage phenotype
MSCs: Mesenchymal stem cells
MTT: 3-(4,5-dimethylthiazol-2-yl)-2,5-diphenyltetrazolium bromide
Ti-Zr: Titanium-zirconium alloy
Ti-6Al-4V: Titanium–6 aluminium–4 vanadium alloy
UVB: Ultraviolet B
VDR: Vitamin D receptor
25(OH)D: 25-hydroxyvitamin D
1α,25(OH)₂D₃: 1α,25-dihydroxyvitamin D₃
